# Association of the 4 g/5 g polymorphism of plasminogen activator inhibitor-1 gene with sudden sensorineural hearing loss. A case control study

**DOI:** 10.1186/1472-6815-12-5

**Published:** 2012-06-06

**Authors:** Seong Ho Cho, Haimei Chen, Il Soo Kim, Chio Yokose, Joseph Kang, David Cho, Chun Cai, Silvia Palma, Micol Busi, Alessandro Martini, Tae J Yoo

**Affiliations:** 1 Division of Allergy-Immunology, Department of Medicine, Northwestern University Feinberg School of Medicine, 676 N. St Clair street #14028, Chicago, IL, 60611, USA; 2 Kyung Hee University, College of Medicine, Seoul, Korea; 3 University of Tennessee, College of Medicine, Memphis, TN, USA; 4 University of Modena, Modena, Italy; 5 University of Ferrara, Ferrara, Italy

**Keywords:** Sudden hearing loss, Plasminogen activator inhibitor-1, 4 G/5 G polymorphism

## Abstract

**Background:**

The 5 G/5 G genotype of PAI-1 polymorphism is linked to decreased plasminogen activator inhibitor-1 (PAI-1) levels and it has been suggested that lower PAI-1 levels may provide protective effects on inflammation, local microcirculatory disturbance, and fibrotic changes, which are likely associated with development of sudden sensorineural hearing loss (SSNHL).

**Methods:**

The association of the 4 G/5 G PAI-1 polymorphism with the development and clinical outcome of SSNHL is evaluated *via* a case control study. 103 patients with SSNHL and 113 age and sex-matched controls were enrolled at University of Ferrara, Italy and hearing loss outcome was measured at least 3 months after the onset of hearing loss. DNA was isolated from peripheral blood using the QIAamp kit and the 4 G/5 G polymorphism in the −675 promoter region was genotyped with an allele-specific PCR. Genotype distribution was tested in patients and compared to controls by chi-square and odd-ratio analysis. The codominant and recessive models were used for the multiple logistic regression analyses of the PAI-1 gene allele.

**Results:**

In this population, 5 G/5 G genotype had a two-time lower frequency in SSNHL patients compared to healthy controls (15.5% vs 30.1%) and was associated with decreased odds compared to 4 G/5 G genotype (OR 0.37, 95% CI 0.19-0.75, *p* = 0.005). In addition, the patients with 5 G/5 G genotype showed a trend of more than 2 times higher ratio of hearing recovery (> 20 dB) after systemic corticosteroid treatment compared to 4 G/5 G genotype (OR 2.3, 95% CI 0.32 - 16.83, *p* = 0.39), suggesting a better clinical outcome.

**Conclusions:**

The 5 G/5 G genotype of PAI-1 may be associated with a reduced risk of SSNHL in the Italian population.

## Background

Sudden sensorineural hearing loss (SSNHL) is defined as a rapid onset sensorineural hearing loss occurring over a 72-hour period with a decrease in hearing of > 30 decibels (dB) affecting at least 3 consecutive frequencies [1]. The majority of patients with SSNHL have no identifiable causes and are thus classified as “idiopathic” [2]. SSNHL affects 5 to 20 persons for each 100,000 individuals annually and can be devastating because they can lose their hearing permanently. The etiology of SSNHL is still unclear although the most recent studies suggest viral infection, vascular impairment, intracochlear membrane rupture, and autoimmune process as possible causes [3,4]. Several studies have been reported on the association between cardiovascular risk factors and SSNHL, showing that high concentrations of cholesterol, fibrinogen, and homocysteine were risk factors [5-9]. Other reports evaluated the association between SSNHL and genetic polymorphisms of thromboembolic factors, mainly factor V Leiden and prothrombin G20210A variant, with controversial results [10-13].

Plasminogen activator inhibitor-1 (PAI-1) is the principal inhibitor of tissue plasminogen activator (tPA) and urokinase-type plasminogen activator (uPA), which actively facilitate plasminogen, and hence fibrinolysis. PAI-1 is a key molecule for thrombus formation and inflammation [14]. Elevation in plasma levels of PAI-1 has been reported to be associated with many diseases, such as cardiovascular diseases [14], stroke [15] and asthma [16-18]. Marcucci et al. [6] also reported that plasma levels of PAI-1 were significantly higher in patients with SSNHL compared to control subjects. One of most probable causes of SSNHL appears to be impaired cochlear blood circulation involving the pathogenic micro-thrombotic mechanism [19,20]. Cochlear function is very sensitive to changes in blood supply which is mainly derived from the labyrinthine artery. Vascular compromise of the cochlea caused by reduced blood flow, vasospasm, thrombosis or embolus, may result in SSNHL [13,21]. Our previous case report demonstrated the effectiveness of fibrinolytic therapy for sudden hearing loss with an improvement of 50 dB using a recombinant tPA, which appeared to improve the microcirculation of the inner ear [20]. These findings suggest that PAI-1 is a possible risk factor and a potential therapeutic target for SSNHL.

The PAI-1 gene has variation in the promoter region on the basis of a single guanosine insertion-deletion (5 G or 4 G) [16]. Previous studies on healthy subjects show the PAI-1 genotype distribution of 4G4G, 4G5G and 5G5G were 36.4%, 50.5% and 12.9% for a German population [11], 27.4%, 47.0%, and 25.6% for an Italian population [22], 28.7%, 42.5%, and 28.7% for a Turkish population [19], and 27.5%, 52.5% and 20.0% for a Spanish population [23] respectively. It has been reported that the subjects with 4 G/4 G have increased plasma PAI-1 levels and the ones with 5 G/5 G have decreased plasma levels, while the ones with 4 G/5 G have intermediate plasma levels [24]. Increased PAI-1 levels facilitate the inhibition of the fibrinolytic system [16], which may impair cochlear blood circulation and thus predispose the development of SSNHL [6,20]. It has been reported that the 4 G/5 G polymorphism of PAI-1 gene is associated with cardiovascular and thromboembolic diseases [16,25]. Rudack, Yildiz, and their coworkers also investigated the association between PAI-1 4 G/5 G polymorphism and SSHNL in the German [11] and Turkish [19] populations although the results were not conclusive.

In this study we investigated the 4 G/5 G polymorphism of PAI-1 gene in the Italian patients with SSNHL to see if this polymorphism is a risk factor in developing SSNHL and can be used as a prognostic indicator for clinical outcome in patients with this disease. We demonstrated a significant contribution of 5 G/5 G polymorphism to lowering the risk of developing SSNHL and a trend of improved hearing recovery in follow up evaluation after treatment of SSNHL.

## Methods

### Patients and controls

One hundred and three patients, 54 females and 49 males (age range, 23–83; female mean age 54.1, male mean age 55.7), were evaluated at the Department of Audiology at the University of Ferrara and Modena with a diagnosis of SSNHL and gave their consent to participate in the study. They were referred to the department from January 2005 to December 2009. All the patients underwent audiological examinations including pure tone audiometry, speech recognition threshold, immittance measurements such as tympanogram and acoustic reflex; other audiometric tests for the differential diagnosis of cochlear vs retrocochlear pathologies were performed in selected cases. Auditory brainstem response (ABR) was always performed except when hearing loss exceeded 80 dB in the acute frequencies. Audiometric inclusion criterion was a decrease in hearing of at least 30 dB in 3 contiguous frequencies. The following scale of hearing loss degree was used: mild, ≥ 30 to < 40 dB hearing loss; moderate, ≥ 40 to < 70 dB hearing loss; severe, ≥ 70 to < 90 dB hearing loss; and profound, ≥ 90 dB hearing loss. Patients did not receive vestibular evaluation because exclusion criteria were the presence of vertigo or suspected Meniere’s disease. Acoustic neuroma was excluded by performing MRI or by serial audiometry in selected cases. Any participant with a history of head trauma was also excluded from the study.

All the subjects provided their informed consent for the study and blood was taken as usual for routine laboratory exams, in the morning before starting the pharmacological therapy. Medical treatment consisted of betamethasone 4 mg i.m. for 3 days and 1.5 mg i.m. for 3–4 days, and oral prednisone was administered for at least 7 days afterwards, depending on clinical evolution. Pure tone audiometry was repeated daily according the history of the patient. We examined follow-up audiometry at least 6 months after the initial episode of hearing loss and considered a hearing improvement more than 20 dB as a significant recovery. The practice guidelines suggest <10 dB changes as no recovery [2]. Therefore we chose 20 dB as a clear cut-off for significant improvement, which would include partial and complete recovery. The age and sex-matched healthy Italian subjects (n = 113) were enrolled in the same geographical area. Control subjects had no history of hearing loss, circulatory or metabolic diseases, or autoimmune disorders. They were healthy clinic staffs and clinic patients with other otolaryngology disorders, such as allergic rhinitis, who gave their consent for the study. All of them had normal hearing.

### DNA extraction

Blood was taken from each patient or control for genomic DNA extraction, which was performed using the QIAamp kit (Qiagen Inc., Valencia, CA) as directed to obtain 3 to 12 μg of DNA from 200 μl whole blood.

### PAI-1 genotyping

The PAI-1 4 G/5 G genotype was analyzed with an allele-specific PCR modified from that of Falk et al. [26], using an alternative forward primer (GTCTGGACACGTGGGGG for the 5 G allele or GTCTGGACACGTGGGGA for the 4 G allele) with a common reverse primer (TGCAGCCAGCCACGTGATTGTCTAG, designed to minimize primer-dimer formation) and a control reverse upstream primer (AAGCTTTTACC ATGGTAACCCCTGGT). The PCR procedure included a hot-start initial step to avoid primer-dimer artifacts. The PCR mixture was subjected to 30-step cycles of 94°C (1 minute), 60°C (1 minute) and 72°C (1 minute). The PCR reaction was performed in a total volume of 25 μl with 0.5 μg of genomic DNA by using C1000^TM^ Thermal Cycler (Bio-Rad Laboratories, Inc., Hercules, CA). The reaction mixture contained 10 mmol/L Tris–HCl (pH 8.0), 2.5 mmol/L MgCl_2_, 200 μmol/L deoxyribonucleoside triphosphates, and 25 pmol of each primer. For each PCR, 2.5 U of Taq polymerase (Promega, Madison, MI) was used. Electrophoresis was performed in 2% agarose with 1 x TAE buffer. The gels were photographed after ethidium bromide staining. As a control of this PCR technique, PCR analysis was performed on DNA samples of known genotypes.

### Statistical analysis

Genotype distribution and allele frequencies were tested in patients and compared to controls by chi-square test (χ^2^) and odd-ratio analysis using GraphPad Prism for Windows version 4.03 (GraphPad Software Inc., San Diego, CA). We also used chi-square test (χ2) and odd-ratio analysis for the association between genotype distribution and hearing outcome. A *p* value of less than 0.05 was considered to be statistically significant. All odds ratios (OR) are given with their 95% confidence interval (CI). In the multiple logistic regression analyses for each PAI-1 gene allele, we used multiple inheritance models, including codominant model (the relative hazard differed between subjects with one minor allele and those with two minor alleles) and recessive model (only subjects with two minor alleles were at increased risk of the disease). 5 G was considered the minor allele in our case.

## Results

**Figure 1:**
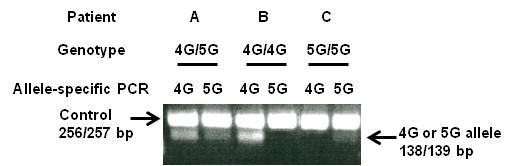
**Gel patterns demonstrating the 4 G and 5 G alleles in the promoter region of the PAI-1 gene.** PCR products with a forward primer for the 4 G allele or the 5 G allele and a control upstream primer are indicated by 4 G or 5 G, respectively; Lanes 1 and 2 for patient **A** (4 G/5 G genotype); Lanes 3 and 4 for patient **B** (4 G/4 G genotype); and Lanes 5 and 6 for patient **C** (5 G/5 G genotype).

A total of 103 SSNHL patients and 113 healthy controls were analyzed for 4 G and 5 G PAI-1 gene alleles. A typical PAI-1 genotyping experiment is shown in Figure 1. The frequencies of allele 4 G were 48.7% in SSNHL and 47.1% in controls, comparable to those for allele 5 G of 51.3% (SSNHL) and 52.9% (controls). The frequencies of genotypes 4 G/5 G, 4 G/4 G, and 5 G/5 G were 61.2%, 23.3%, and 15.5%, respectively, in SSNHL, and 44.2%, 25.7%, and 30.1% in controls (Table 1). The prevalence of 5 G/5 G genotype in the SSNHL patients (15.5%) was two times lower than that in control (30.1%). The 5 G/5 G genotype appeared to have the risk effect three times lower than that of the 4 G/5 G genotype (OR 0.37, 95% CI 0.19-0.75, *p* < 0.005), which is the most prevalent genotype. When we compared the 5 G/5 G genotype with combined non-5 G/5 G genotypes (4 G/5 G + 4 G/4 G), the 5 G/5 G genotype had two times lower risk of developing SSNHL than non-5 G/5 G genotypes. However, the 4 G/4 G genotype did not have significantly increased risk compared to the 4 G/5 G genotype.

**Table 1:** Genotype distribution of PAI-1 polymorphism in SSNHL and controls

Model	Genotype	Controls, n = 113 (Frequency)	SSNHL, n = 103 (Frequency)	OR	95 % CI	χ^2^	*p* value
Co-dominant	4 G/5 G	50 (44.2 %)	63 (61.2 %)	1			
	4 G/4 G	29 (25.7 %)	24 (23.3 %)	0.66	0.34 – 1.27	1.59	0.210
	5 G/5 G	34 (30.1 %)	16 (15.5 %)	0.37	0.19 - 0.75	7.83	**0.005**
Recessive	4 G/4 G-4 G/5 G	79 (69.9 %)	87 (84.5 %)	1			
	5 G/5 G	34 (30.1 %)	16 (15.5 %)	0.43	0.22 - 0.83	6.42	**0.011**

*SSNHL* sudden sensorineural hearing loss; Odds ratios (OR) with 95 % confidence intervals (CI) and results (*p* values) of χ^2^ analysis (chi-square test) were calculated for genotype frequencies compared with control.

We further examined to see if the polymorphism of PAI-1 gene is associated with clinical outcome in patients with SSNHL (Table 2). We were able to obtain follow-up pure tone audiometry from only 34 patients. Of these patients, SSNHL was unilateral in 33 patients and bilateral in 1. Among the unilateral cases, the degree of SSNHL was mild in 6 patients, moderate in 15, severe in 8, and profound in 4; one bilateral case was moderate degree in both side. Among these SSNHL patients, patients with 5 G/5 G genotype showed a tendency to have better outcome with 60% of patients having > 20 dB recovery after treatment, while patients with 4 G/5 G and 4 G/4 G genotypes had only 39.1% and 33.3% recovery rates (> 20 dB improvement), respectively. The genotype 5 G/5 G appeared to have a recovery effect 2.3 times higher than that of 4 G/5 G (OR 2.33; 95% CI 0.32 - 16.83). However, this finding of better clinical outcome in patients with the 5 G/5 G genotype was not statistically significant due to a relatively small number of patients who had received the available follow-up pure tone audiometry.

**Table 2:** Significant hearing improvement (> 20 dB) in PAI-1 polymorphism

Genotype	4 G/5 G, n = 23 (Frequency)	5 G/5 G, n = 5 (Frequency)	4 G/4 G, n = 6 (Frequency)
No improvement	14 (60.9%)	2 (40.0%)	4 (66.7%)
Improvement	9 (39.1%)	3 (60.0%)	2 (33.3%)
OR (95% CI)	1	2.33 (0.32 - 16.83)	0.78 (0.12 - 5.17)
χ^2^; *p* value		0.73; 0.39	0.068; 0.79

*OR* odd ratio, *CI* confidence interval.

## Discussion

One of most probable mechanisms of SSNHL appears to be impaired cochlear blood circulation involving the pathogenic micro-thrombotic mechanism [20]. There are precedent polymorphism studies on the roles of various prothrombotic risk factors in SSNHL, including GPIa C807T [27], FV 1691 G-A [11,28], MTHFR 677 C-T [12,19], and G20210A [10,29]. It has been known that elevated plasma levels of PAI-1 are associated with SSNHL [6], but the role of the PAI-1 is controversial [6,11]. In this study, we found that the 5 G/5 G genotype of the PAI-1 gene was associated with reduced risk of developing SSNHL.

To investigate the potential contribution of polymorphism within the PAI-1 gene to the development of SSNHL, we recruited SSNHL patients and control subjects from Ferrara and Modena, Italy, from a white, homogeneous population. In this population, we found the frequencies of 5 G allele (51.3%, 52.9%) had no significant difference from those of 4 G allele (48.7%, 47.1%) either in patients or in controls. However, the frequency of the 5 G/5 G genotype was two times lower in the SSNHL group (15.5%) compared to that in the control group (30.2%). This 5 G/5 G genotype showed 2–3 times lower risk effect than 4 G/4 G and 4 G/5 G. In this study, we found the 4 G/4 G genotype had no significant risk ratio in developing SSNHL. However, the 5 G/5 G genotype appeared to have a protective effect against developing SSNHL. Our findings are supported by several previous reports that the patients with 5 G/5 G genotype are known to have lower plasma levels of PAI-1 compared to those with 4 G/4 G [16,24], and lower PAI-1 level can provide protective effects on inflammation, local microcirculatory disturbance, and fibrotic changes, which are likely associated with developing SSNHL [4,6,30]. Our data showing the significance of the 5 G/5 G genotype are different from a previous study on German patients with SSNHL [11], where the 5 G/5 G genotype in the experimental group had no significant difference in frequency compared to the controls. Notably, their study showed the control genotype distribution were 36.4% (4G4G), 50.5% (4G5G), and 12.9% (5G5G), significantly different from our control study with Italian population (25.7%, 44.2%, and 30.1%). Additionally, their study elected only severe SSNHL patients with a loss of 60 dB or more. In contrast, our study recruited patients with a hearing loss of 30 dB or more. It is not clear whether the discrepancy between these two studies is due to different population (Italian vs German) or different severity of the disease.

It has been known that there is no effective treatment of SSNHL other than systemic corticosteroids which is generally used but has limitation to some patients [2]. Our study suggests that lowering plasma levels of PAI-1 may be a strategy to prevent SSNHL, especially in people who are likely to have high plasma levels of PAI-1, such as subjects who are obese, diabetic, or smokers [16,18]. Our study also suggests that the patients with the 5 G/5 G genotype have a tendency of 2–3 times higher ratio of hearing recovery (> 20 dB) compared to those with the 4 G/4 G and 4 G/5 G genotypes, although it was not statistically significant. We defined a change in hearing of > 20 dB as significant improvement, although practice guidelines usually suggest >10 dB improvement as significant, which includes both partial and complete recovery. We had a limited number of long-term follow-up audiometry results, which resulted in a lack of power in our analysis. Collecting more data on long-term follow-up audiometry would provide better insight into the influence of 4 G/5 G genotype on the clinical outcomes of SSNHL. We previously reported a case which showed significant improvement of hearing (> 50 dB) with recombinant tPA treatment two years after the development of SSNHL [20]. This case is an interesting observation that may suggest a link between fibrinolytic treatment and clinical outcome in patients with SSNHL. However, it is too early to suggest tPA use as an alternative treatment for SSNHL.

## Conclusions

This study suggests that the individuals with the 5 G/5 G genotype of PAI-1 have less risk of developing SSNHL, and the 5 G/5 G genotype may function as a prognostic factor in recovery from SSNHL. This result may be of clinical significance in diagnosis, treatment, and prognosis for SSNHL patients and may provide a new therapeutic strategy for SSNHL.

## Abbreviations

SSNHL, Sudden sensorineural hearing loss; PAI-1, Plasminogen activator inhibitor-1; tPA, Tissue plasminogen activator; uPA, Urokinase-type plasminogen activator.

## Competing interests

Authors declare that there is no financial or non-financial competing interests in relation to this manuscript.

## Authors’ contributions

SC, SP, MB, AM and TY conceived of the study, and participated in its design and coordination and helped to draft the manuscript. HC, CY, DC and JK involved in drafting the manuscript or revising it critically for important intellectual content and performed the statistical analysis. IK and CC carried out the molecular genetic studies, participated in drafted the manuscript. All authors read and approved the final manuscript.

## Pre-publication history

The pre-publication history for this paper can be accessed here:

http://www.biomedcentral.com/1472-6815/12/5/prepub
